# Supplementary material for: Mutant mice with rod-specific VPS35 deletion exhibit retinal α-synuclein pathology-associated degeneration
Source: Nat Commun. 2024 Jul 23;15:5970. doi: 10.1038/s41467-024-50189-0 (PMC11266608; doi:10.1038/s41467-024-50189-0)
Supplement: Supplementary file 1 — Supplementary Information [file 41467_2024_50189_MOESM1_ESM.pdf]

SUPPLEMENTARY FIGURES

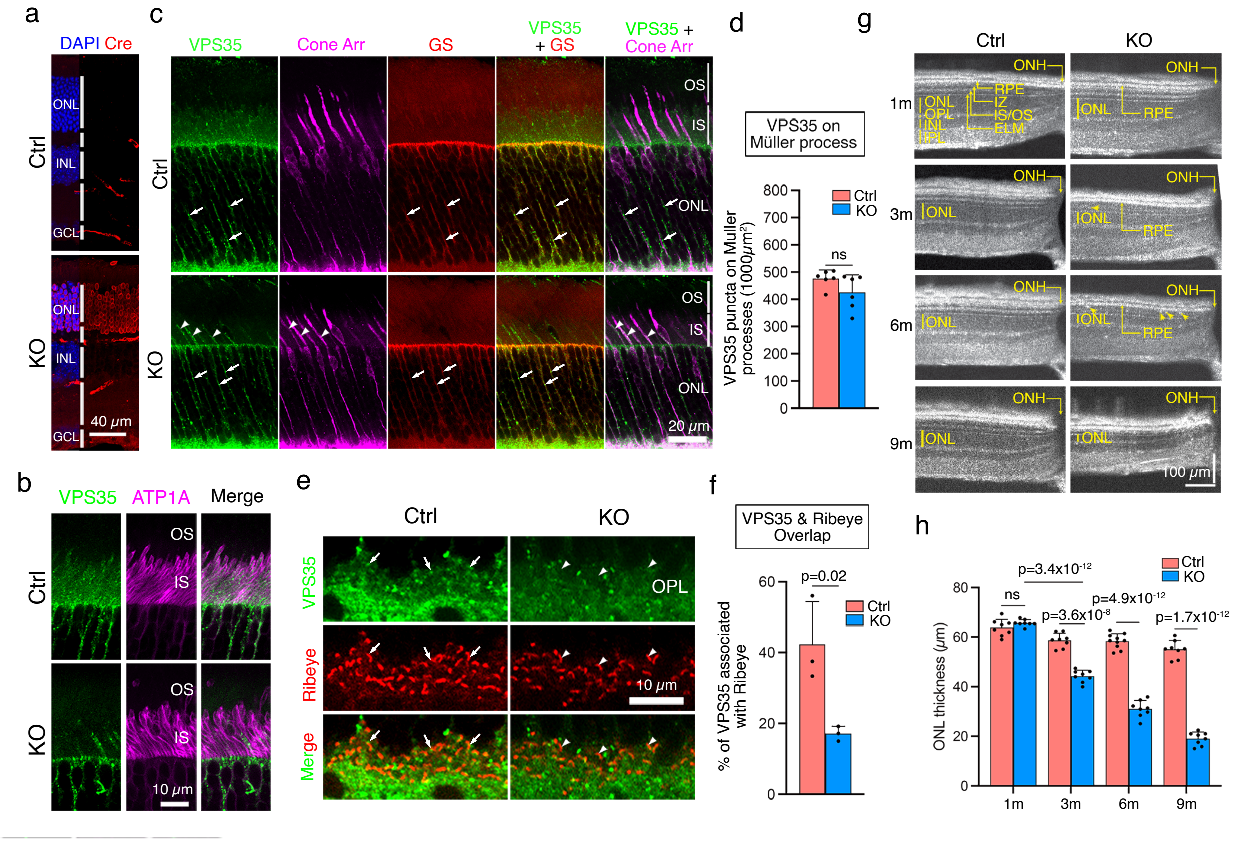

Supplementary Figure 1. Supporting data of Fig. 1

(**a**) 1-month-old mouse retinas stained with Cre (red) and DAPI nuclear dye. Representative images of 3 experiments are shown.

(**b-f**) Retinal staining of 2-month-old Ctrl and KO for VPS35 together with various markers. (b) The co-staining with ATP1A, a photoreceptor IS plasma membrane marker, highlights the IS-expression of VPS35 in Ctrl and loss of the majority of IS signal of VPS35. (c) The co-staining with cone arrestin (Arr), a cone marker, reveals residual IS signals of VPS35 in KO are cones (arrowheads). The co-staining with glutamine synthase (GS), which marks Müller glial cells, demonstrates the similar expression of VPS35-labled puncta in Müller glia processes spanning through the ONL region of both Ctrl and KO mice (arrows). The merged views of between VPS35 and GS or cone arrestin are also shown. (d) shows the number of VPS35 puncta in 1,000 um^2 of Müller glia processes in ONL region. Mean ± SD, N=3 mice in each group. Two-way Student's *t*-test**.** Representative images of 3 experiments are shown.
(e) The co-staining with Ribeye highlights the presence of VPS35 on Ribeye-labeled rod terminals in Ctrl (arrows) and loss of VPS35 in Ribeye+ rod terminals in KO (arrowheads). (f) show the comparison between the fraction of the Ribeye signals in rod terminals that also had VPS35 signals between KO and Ctrl. Mean ± SD, N=3 mice in each group. Two-way Student's *t*-test**.** Two independent repeats with similar results.
(**g**) Representative fundus SD-OCT images show the cross-section views of age-matched Ctrl and KO mouse retinas in real time. Yellow arrows mark the hyperreflective foci underneath the RPE layers, likely representing the microglia infiltrated to the subretinal space. Bars = 100 mm. (h) Bar graphs show the ONL thickness in age-matched Ctrl and KO, measured ~400 mm away from optical nerve head (ONH) in SD-OCT images using InSight Segmentation software. IZ: interdigit zone (RPE/OS interface). ELM: external limiting membrane; ONH: optical nerve head. Mean ± SD, N is more than 8 eyes in each group. Two-way Student's *t*-test**.** Source data of (d, f, h) are provided as a Source Data file.

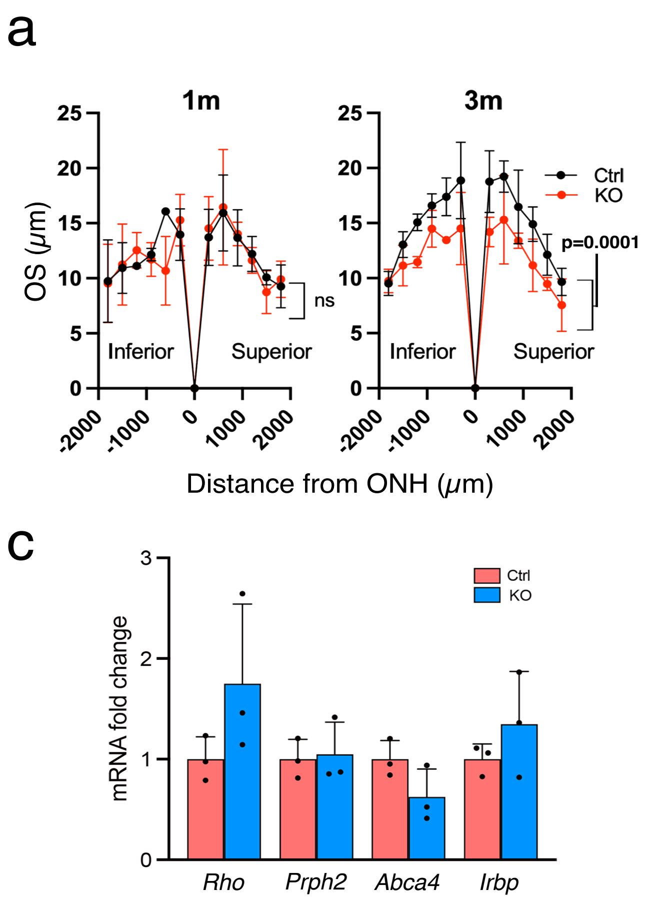

Supplementary Figure 2. Supporting data of Fig. 3

(**a**) Comparing the OS length of 1- and 3-month-old Ctrl and KO. Quantification of the OS length across the superior and inferior retinal hemispheres in relationship with the distances (μm) to the optical nerve head (considered as 0). Mean ± SD. N=3 mice in each group. * $p < 0.05$, ** $p < 0.01$, *** $p < 0.001$. Two-way ANOVA test.
(**b**) Photoreceptor layer staining of OS protein peripherin2 (PRPH2) was evidently decreased in KO (vs. Ctrl). DAPI labeled the ONL. Representative images of 3 experiments are shown.
(**c**) Realtime qPCR results show none of the mRNAs of indicated OS molecules tested (normalized with GAPDH mRNA) are significantly different between 3-month-old KO and Ctrl. Mean ± SD, N=3 mice in each group. Two-way Student's *t*-test**.** Two independent repeats with similar results. Source data of (a, c) are provided as a Source Data file.

a

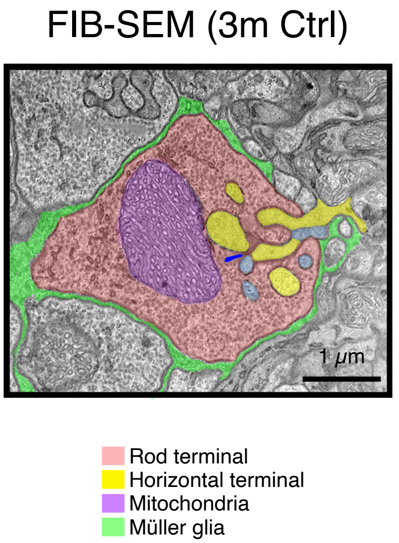

b

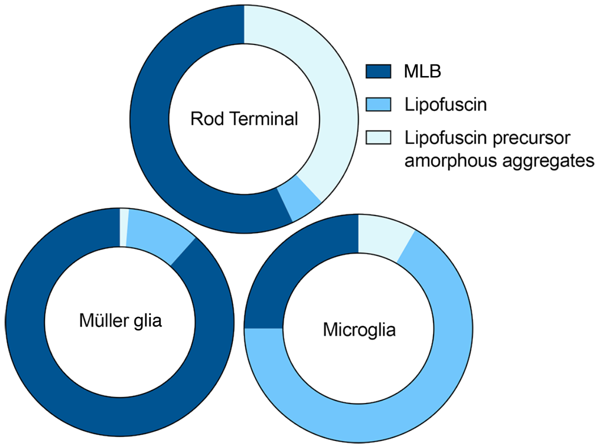

c

Count/mm^3	Rod Terminal	Müller glia	Microglia
Amorphous aggregate	5.64	0.16	0.88
Lipofuscin	0.90	2.36	3.99
MLB	8.18	14.46	1.62

Supplementary Figure 3. Supporting data of Fig. 4.

(a) FIB-SEM image of 3-month-old Ctrl rod terminal had no visible amorphous aggregates, lipofuscins, or MLBs. The colors that mark different subcellular structures are also shown. Representative images of two independent repeats are shown.

(**b, c**) (b) Survey of FIB-SEM stacked images of the OPL region in 3-month-old KO. (c) The distribution percentage of different types of wastes (lipofuscins, amorphous aggregates, and MLBs) (b) and their density (counts per mm^3) in different types of cells (rod terminals, microglia, and Müller glia). Source data of (c), obtained from 3 images stacks of N=2 mice, are provided as a Source Data file.

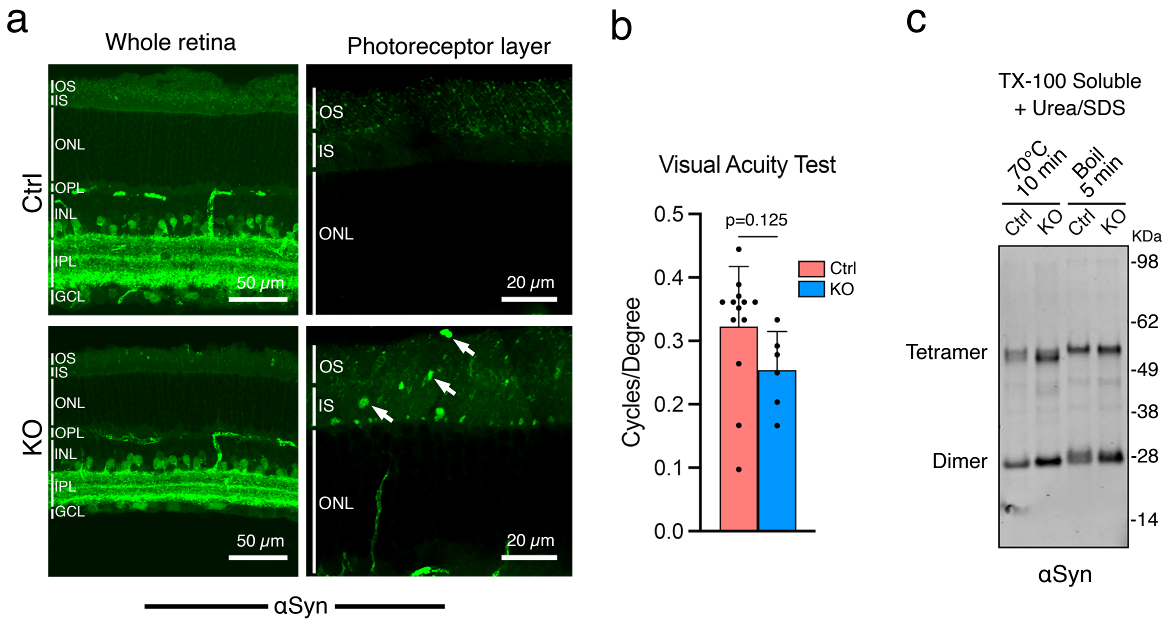

Supplementary Figure 4. Supporting data of Fig. 5.

(a) Zoom-out and zoom-in views show the expression pattern of αSyn in the entire retina (Left) and in the photoreceptor layer (right) of 3-month-olds. Large αSyn-labeled granules were distinctly visible in the IS-OS region of KO (arrows). Representative images of 3 independent repeats are shown.
(b) Visual acuity tests show the 2~3-month-old KO mice had reduced spatial frequency (cycles/degree) at 100% contrast and rotating velocity of 12°/s. Mean ± SD, N=13 (Ctrl) and N=6 (KO). Three independent measurements each animal. Two-way Student's *t*-test**.**
(c) TX-100 soluble retinal lysates mixed with 8M urea/2% SDS (1:10 volume: volume) were denatured in sample buffer and heated at 70°C for 10 min or boiled for 5 min before electrophoresis and immunoblotted for αSyn. Two independent repeats with similar results.
Source data of (b) are provided as a Source Data file.

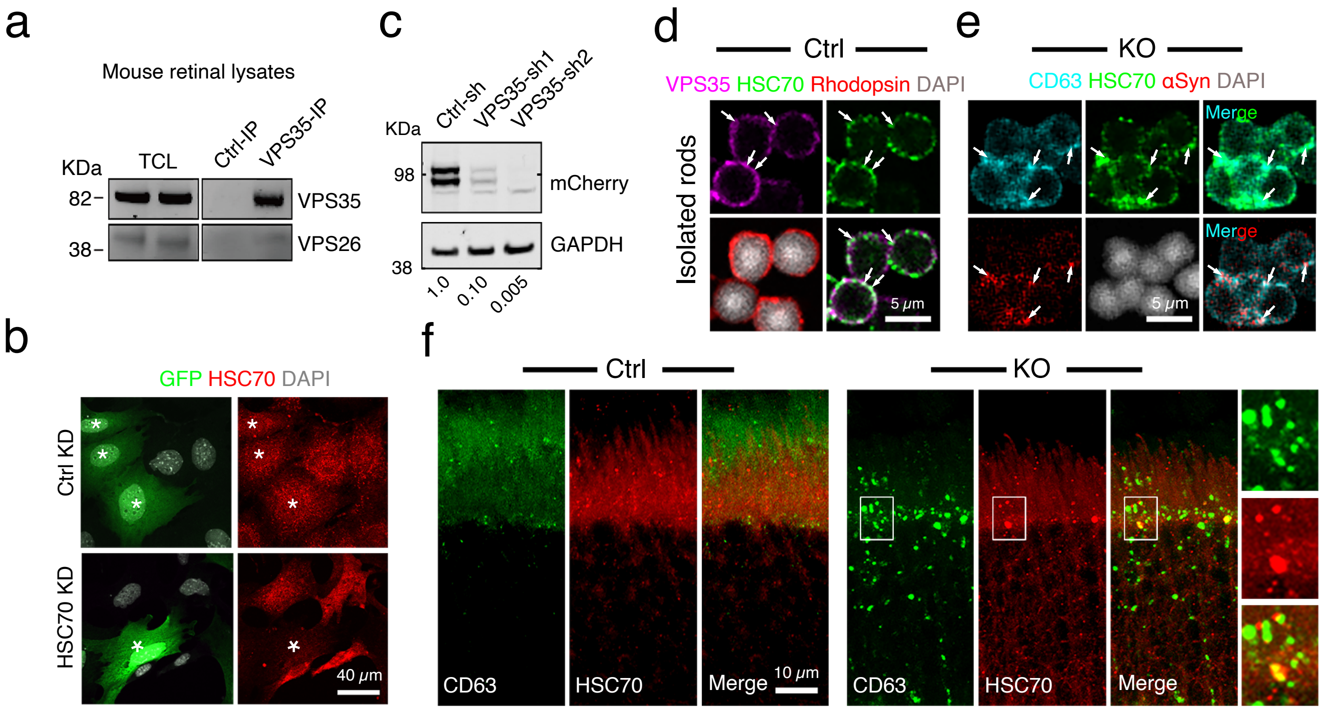

Supplementary Figure 5. Supporting data of Fig. 6

(**a**) Protein blots containing total retinal lysates (TCL) of 1.5-month-old C57BL/6J mice or immunoprecipitants (IP), pulled down by Ctrl or VPS35 antibody followed by the elution with the antigenic peptides that raised VPS35 antibody, were probed for VPS35 and VPS26. Representative blots of three independent repeats are shown.
(**b**) HSC70 antibody immunospecificity was demonstrated by the selective reduced HSC70 staining in "green" (asterisk) 661W cells transfected with plasmid encoding HSC70-shRNA-IRES-GFP (HSC70 KD), but not Ctrl-shRNA-IRES-GFP (Ctrl KD). Representative images of three independent repeats are shown.
(**c**) Validation of VPS35-shRNA-mediated knockdown effect. Protein blots containing 293T cells transfected with mCherry-tagged mouse VPS35 and VPS35-shRNA (sh1, sh2 are two shRNAs against two different target sites of mouse *Vps35* gene) probed for mCherry and GAPDH antibodies are shown. The relative signal of mCherry-VPS35 normalized using GAPDH (considering Ctrl as 1) was shown. Representative blots of two independent repeats are shown.
(**d. e**) Rod isolated from 1.5-month-old Ctrl (d) and KO (e) mice, exhibiting characteristic nuclear profile [1] and expressed rhodopsin, were co-stained with HSC70, DAPI, together with VPS35 (d) or αSyn (e). Arrows (in d) mark the overlapping signals between VPS35 and HSC70 in Ctrl rods. Arrows (in e) mark the co-enrichment of HSC70, CD63 and αSyn in KO rods. Primary rods exhibit primarily perikarya as they lost their OS and IS during dissociation processes. Representative images of two independent repeats.
(**f**) Retinal staining of 2.5-month-old Ctrl and KO for HSC70 and CD63. Zoom-in views of the boxed areas highlight the bright HSC70 staining in enlarged CD63-labeled LEs. Representative images of N=3 mice and two independent repeats are shown.

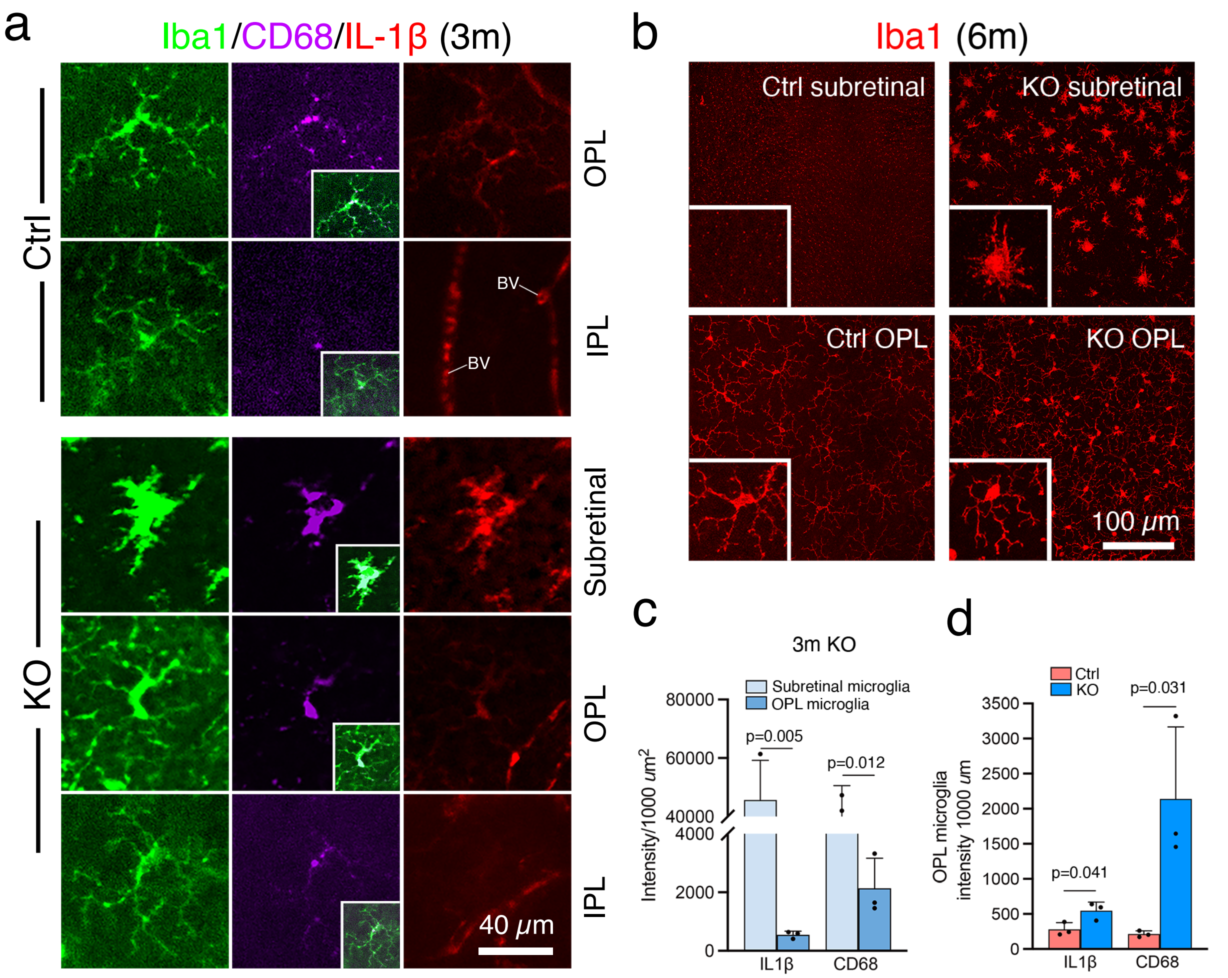

Supplementary Figure 6. Supporting data of Fig. 7

(a) Representative *en face* views of IL-1β (red), CD68 (magenta), and Iba1 (green) staining in different layers of 3-month-old Ctrl and KO flat retinal mounts. Insets show the merged image of Iba1 and CD68. The average measurements of the perimeter and radius of Iba1-labeled microglia are shown in Fig. 7b. The quantification of IL-1β and CD68 is shown below in (d). The non-specific staining of blood vessels (BV) was due to anti-mouse secondary antibodies. IL-1β and CD68 were undetectable in the IPL microglia of both Ctrl and KO. Representative images of N=3; two independent repeats are shown.
(b) Representative *en face* views of Iba1-stained microglia (red) in subretinal and OPL layers of 6-month-old mouse retinal flat mounts. Microglia are found in the subretinal layer of KO but not Ctrl. Microglia in KO (vs. Ctrl) in the OPL had less visible ramification. Representative images of N=3; two independent repeats are shown.
(c) Relative expression level of IL-1β and CD68 in Iba1-labeled microglia distributed in the subretinal and OPL regions of 3-month-old KO. Mean ± SD, N=3 mice each group. Two-way Student's *t*-test.
(d) Relative expression of IL-1β and CD68 detected in the Iba1-labeled microglia distributed in the OPL of 3-month-old Ctrl and KO. Mean ± SD, N=3 mice each group. Two-way Student's *t*-test. Source data of (c, d) are provided as a Source Data file.

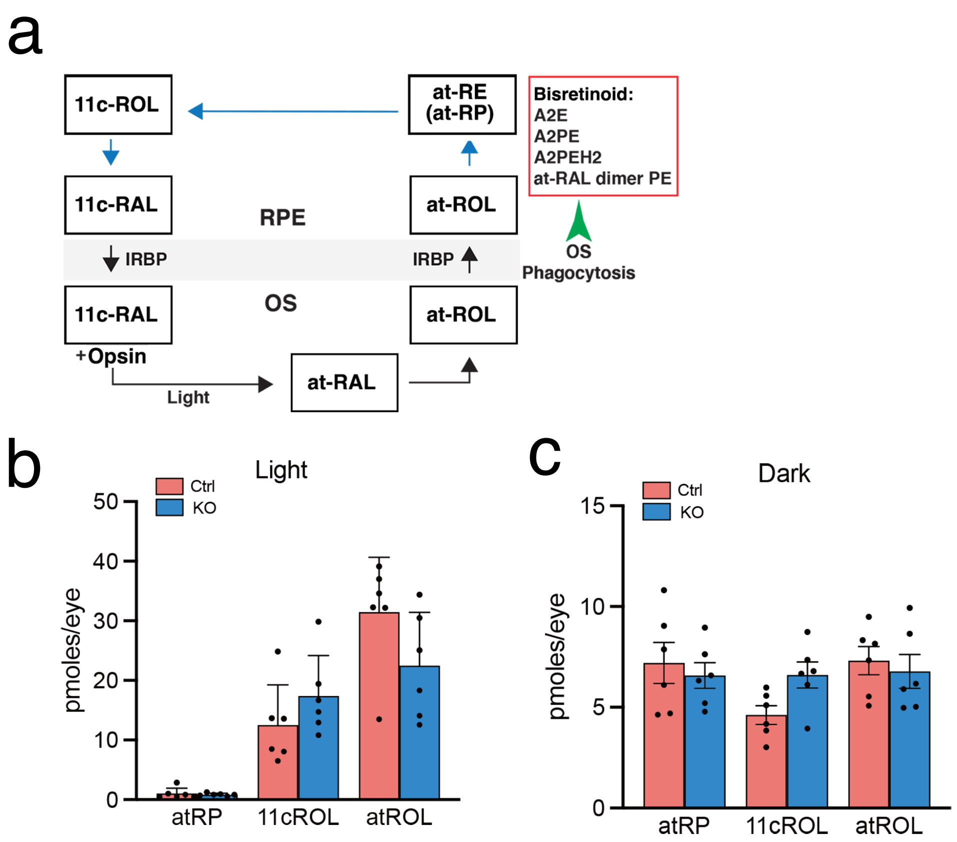

Supplementary Figure 7. Supporting data of Fig. 8.

(a) A diagram illustrates the retinoids produced in the visual cycle.
(b, c) Retinoid profiling. Histograms show the expression level of indicated retinoid species (1-month-old, dark- and light-adapted mouse eyes; N=6 eyes from 3 mice of each genotype). Mean ± SEM and P-values are shown. Two-way Student's *t*-test. Two independent repeats with similar results. Source data of (b, c) are provided as a Source Data file.

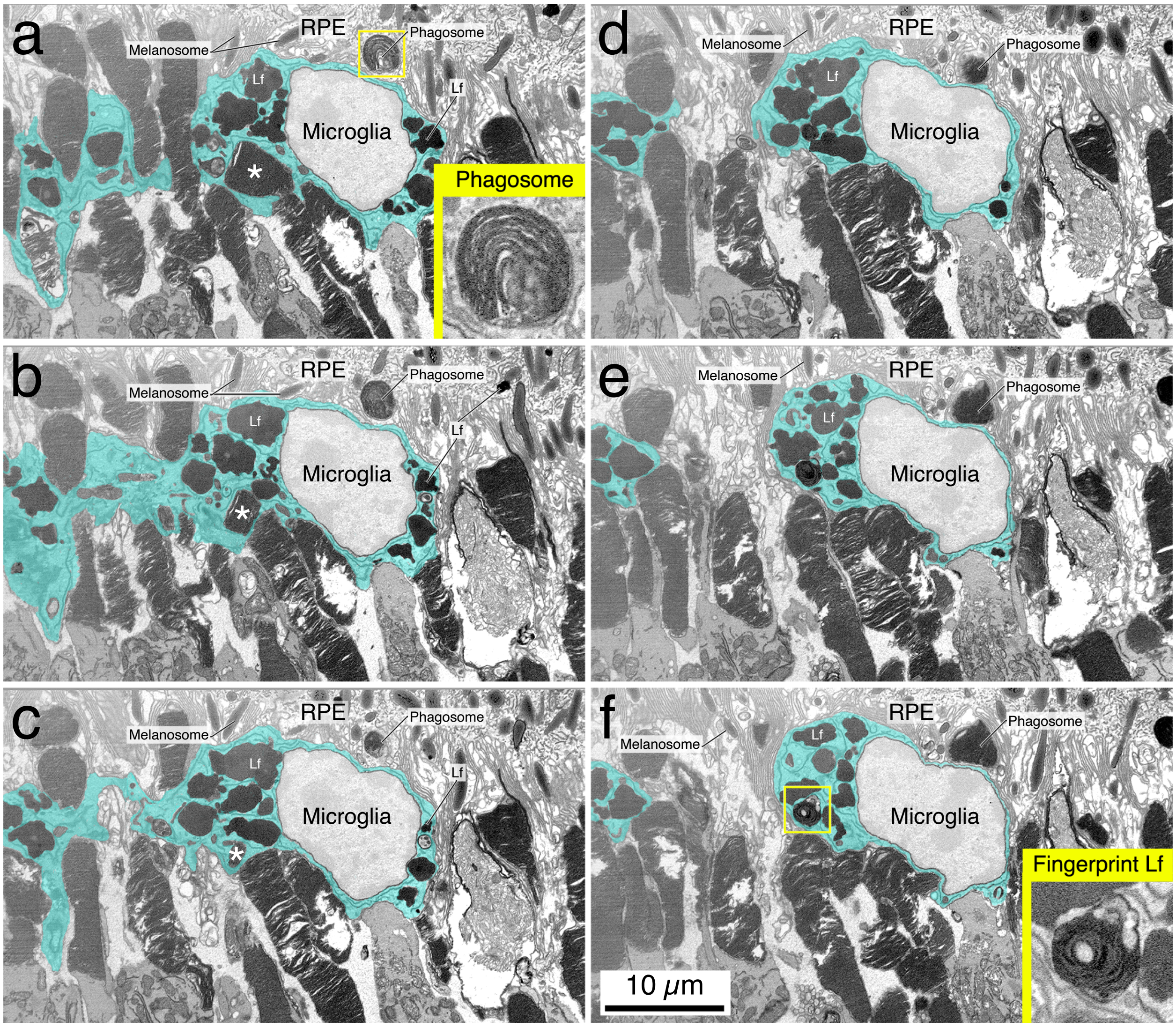

Supplementary Figure 8. Supporting data of Fig. 7h.

(**a-f**) A collage of 6 FIB-SEM images (z stack: 200-nm) of 3-month-old KO mice shows a subretinal microglia (shaded in blue) with a large cell body surrounded by the RPE and several OS. Inset of (a) highlights a phagophore containing OS disc membranous whorls founding in RPE cells. Inset of (f) highlights a fingerprint-pattern lipofuscins (Lf) found in subretinal microglia cells. Inspection of consecutive views can readily distinguish OS disc membranes, phagophores, and fingerprint lipofuscins. In a given single *en-face* view, an OS tip might be mistaken as an internalized phagosome in the subretinal microglia cell (Asterisks in a-c). Representative images of N=2 mice, two independent repeats are shown.

Original Fig.3c

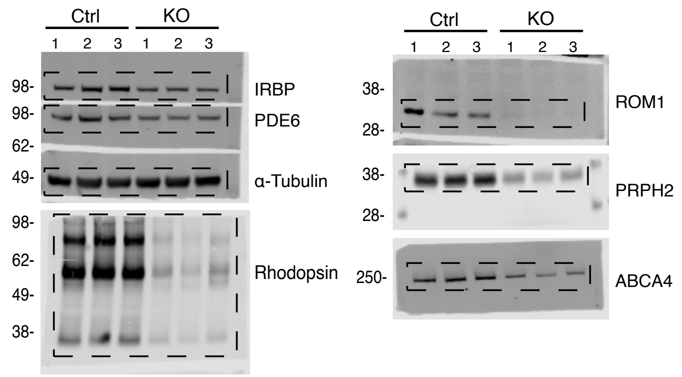

Original Fig.3g

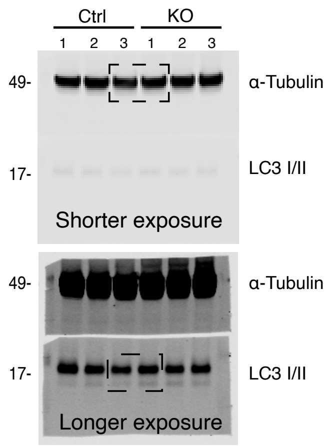

Original Fig.5e (right)

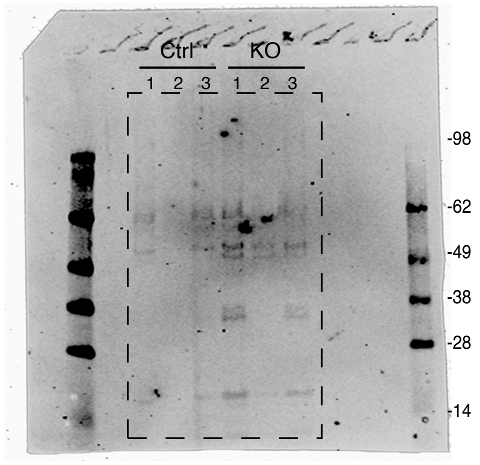

Original Fig.6d

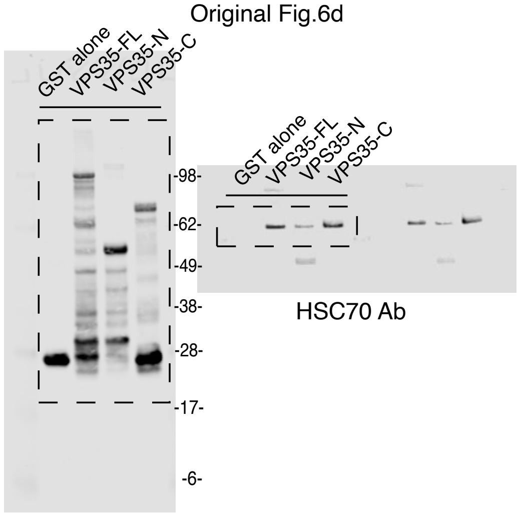

Original Fig.5e (left)

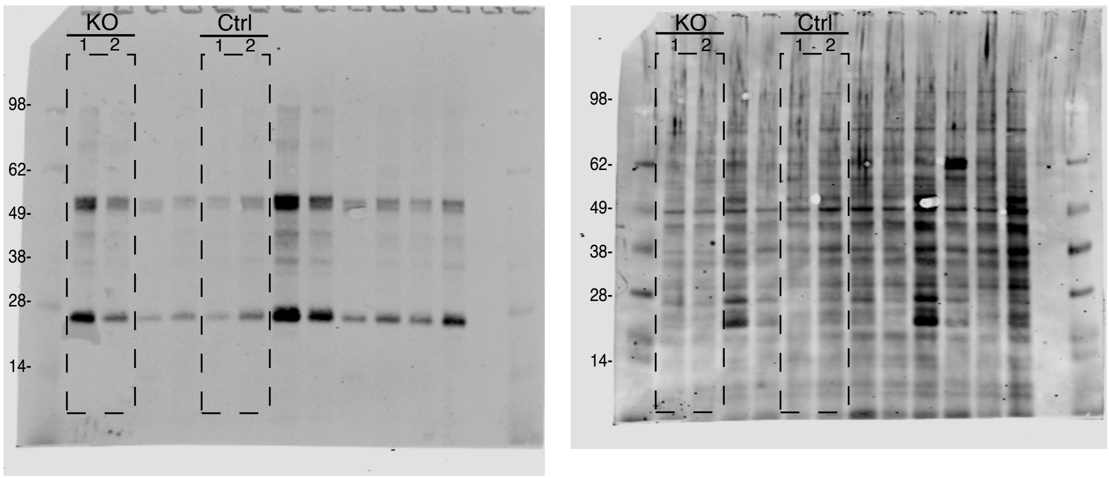

Supplementary Figure 9. Original blots of Figures 3c, 3g, 5e, and 6d.

Original Fig.S4c

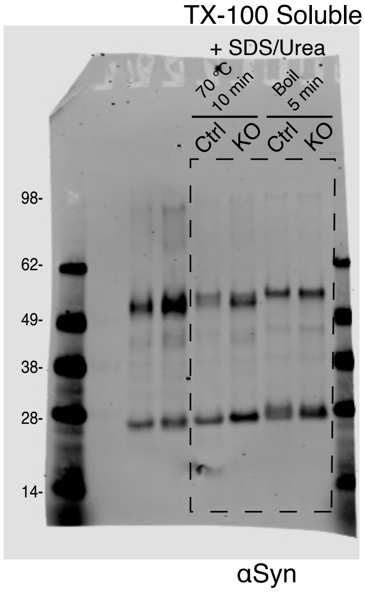

Original Fig.S5a

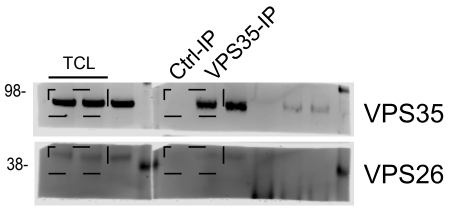

Original Fig.S5c

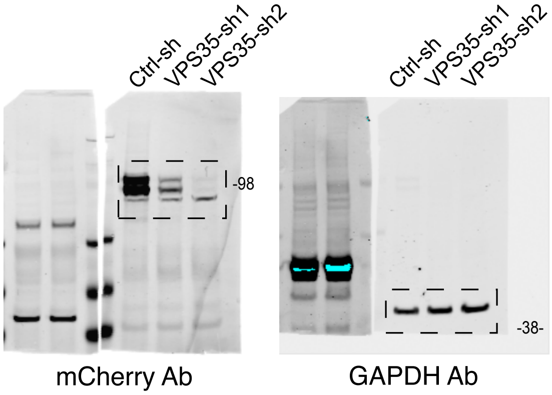

Supplementary Figure 10. Original blots of Supplementary Figures 4c, 5a, and 5c.

Supplementary Table 1. Summary of proteomics dataset of VPS35 immunoprecipitants obtained from three independent experiments (Exp). Previously reported HSC70 substrates and HSC70 interacting proteins are also listed. Source data are provided as a Source Data file.

Protein name		Accession Number	Total number of peptide sequences associated with the protein group			Published Hsc70 substrate Y: Yes	HSC70 interacting
	Full Name		Exp. 1	Exp. 2	Exp. 3		
Acta1	Actin, alpha skeletal muscle	P68134	8	7			
Actb	Actin, cytoplasmic 1	P60710	14	12		Y pubmed	
Actg1	Actin, cytoplasmic 2	G3UZ07		12	4	Y pubmed	
Actn4	Alpha-actinin-4	P57780	1	1		Y pubmed	Co-fractionation
Actr1a	Alpha-centractin	P61164		1			
Ahcy	Adenosylhomocysteinase	P50247	3				
Aldoa	Fructose-bisphosphate aldolase A	P05064	2				
Anxa1	Annexin A1	P10107	3	1	3		
Anxa2	Annexin A2	P07356	9	9	3		
Anxa4	Annexin A4	P97429	2				
Ap2b1	AP-2 complex subunit beta	Q9DBG3	1				
Aqp4	Aquaporin-4	P55088	5	3	2		
Arf3	ADP-ribosylation factor 3	P61205					
Arg1	Arginase-1	Q61176		2	1		
Arhgef28	Rho guanine nucleotide exchange factor	P97433		1			
Arpc3	Actin-related protein 2/3 complex subunit 3	Q9JM76	1				
Atp1a3	Sodium/potassium-transporting ATPase	Q6PIC6			1		
Atp5a1	ATP synthase subunit alpha, mitochondrial	Q03265		10	4	Y	BioGRID
Atp5b	ATP synthase subunit beta, mitochondrial	P56480		8		Y	BioGRID
Bdp1	Transcription factor TFIIIB component B	Q571C7	1				
Blmh	Bleomycin hydrolase	Q8R016		8			
Bsn	Protein bassoon	O88737			1		
Cacna1e	Voltage-dependent R-type calcium	Q61290		1			
Calml3	Calmodulin-like protein 3	Q9D6P8	1				
Cap1	Adenylyl cyclase-associated protein 1	P40124	1				
Capg	Macrophage-capping protein	P24452	1			Y，pubmed	Affinity Capture-
Capza1	F-actin-capping protein subunit alpha-1	P47753	1			Y，pubmed	Affinity Capture-
Capzb	F-actin-capping protein subunit beta	P47757	3			Y，pubmed	Affinity Capture-
Casc5	Protein CASC5	Q66JQ7	1		1		
Cat	Catalase	P24270	3	3	2		
Ccdc30	Coiled-Coil Domain Containing 30	A8WHU1			1		
Ccdc73	Coiled-coil domain-containing protein 73	Q8CDM4	1				
Cenpf	Centromere Protein F	E9Q3P4		1			
Cfap221	Cilia- and flagella-associated protein 221	A9Q751	1				
Cfl1	Cofilin-1;Cofilin-2	P18760	2				
Col1a1	Collagen alpha-1(I) chain	P11087			2		
Col1a2	Collagen alpha-2(I) chain	Q01149		1	1		
Copb2	Coatomer subunit beta	O55029	1	1			
Cpa4	Carboxypeptidase A4	Q6P8K8	1				
Crmp1	Dihydropyrimidinase-related protein 1	P97427	2		2		
Crocc	Ciliary Rootlet Coiled-Coil, Rootletin	Q8CJ40		1			
Cryaa	Alpha-crystallin A chain	P24622-2		8	4		
Cryab	Alpha-crystallin B chain	P23927		3	1		
Cryba1	Crystallin Beta A1	Q9QXC6		3	5		
Cryba2	Beta-crystallin A2	A0A087WQ		1	1		
Crybb2	Beta-crystallin B2	P62696		3	1		
Ctbp1	C-terminal-binding protein 1	O88712			5		
Ctbp2	C-terminal-binding protein 2	P56546-2	2	6	8	Y	BioGRID
Ctsd	Cathepsin D	P18242	1	1			
Ddx4	Probable ATP-dependent RNA helicase	Q61496			1		
Dpysl2	Dihydropyrimidinase-related protein 2	O08553		2	2	Y	BioGRID
Dpysl3	Dihydropyrimidinase-related protein 3	Q62188			1	Y	BioGRID
Dsg1a	Desmoglein-1-alpha	Q61495	4	6	2		
Dsp	Desmoplakin	E9Q557	82	59	24	Y	BioGRID
Dstn	Destrin	Q9R0P5			1		
Edil3	EGF-like repeat and discoidin I-like	O35474			2		
Eef1a1	Elongation factor 1-alpha 1	P10126	5	4	1	Y	BioGRID
Eef1g	Elongation factor 1-gamma	Q9D8N0	3	2		Y	BioGRID
Eef2	Elongation factor 2	P58252	7	3		Y	BioGRID
Eif4a1	Eukaryotic initiation factor 4A-I	P60843	1	3		Y	BioGRID

Eif6	Eukaryotic translation initiation factor 6	O55135		2			
Eml1	Echinoderm microtubule-associated	Q05BC3		2	3		
Eno1	Alpha-enolase	P17182	6	3	1	Y	BioGRID
Eppk1	Epiplakin	Q8R0W0	5				
Erh	Enhancer of rudimentary homolog	P84089	1		1	Y	BioGRID
Esyt1	Extended synaptotagmin-1	Q3U7R1	1				
Ezr	Ezrin	P26040		3		Y	PMID: 19737422
Fabp5	Fatty acid-binding protein, epidermal	Q05816	1	1	1		
Fam192a	Protein FAM192A	Q91WE2			1		
Fgg	Fibrinogen gamma chain	Q8VCM7	1				
Flna	Filamin-A	Q8BTM8	2			Y	BioGRID
Flnb	Filamin-B	Q80X90	1			Y	BioGRID
Gapdh	Glyceraldehyde-3-phosphate	P16858	5	13	5	Y	BioGRID
Gcn1l1	GCN1 Activator Of EIF2AK4	E9PVA8	1			Y	BioGRID
Gdi2	Rab GDP dissociation inhibitor beta;Rab	Q61598		2		Y	BioGRID
Ggct	Gamma-glutamylcyclotransferase	Q9D7X8		1			
Gimap8	GTPase IMAP family member 8	Q75N62	1				
Glul	Glutamine synthetase	P15105	1	4	1		
Gnao1	Guanine nucleotide-binding protein G(o)	P18872		4			
Gnat1	Guanine nucleotide-binding protein G(t)	P20612		6	4		
Gnb2l1	Guanine nucleotide-binding protein subunit	P68040	2			Y	BioGRID
Gsdma	Gasdermin-A	Q9EST1	3	3	3		
H2afx	Histone H2AX	P27661		2			
Hal	Histidine ammonia-lyase	P35492	4	4			
Hars	Histidine--tRNA ligase, cytoplasmic	Q61035	3	1		Y	BioGRID
Hba	Hemoglobin Subunit Alpha	P01942			2	Y	BioGRID
Hbbt1	Hemoglobin subunit beta-1	A8DUK4		2	2		
Hist1h1e	Histone H1.4	P43274			5	Y	BioGRID
Hist1h2af	Histone H2A type 1-F	A0A0N4SV	2				
Hist1h2bc	Histone H2B type 1-C/E/G	Q6ZWY9	1	3	2		
Hist1h3a	Histone H3.1	P68433	1	2	1	Y	BioGRID
Hist1h4a	Histone H4	P62806	2	6	2	Y	BioGRID
Hist2h2aa	Histone H2A type 2-A	Q6GSS7			1		
Hmgb3	High mobility group protein B3	O54879		1			
Hnrnph1	Heterogeneous nuclear ribonucleoprotein	O35737	1	2			
Hnrnpk	Heterogeneous nuclear ribonucleoprotein	P61979		2		Y	BioGRID
Hsp90aa1	Heat shock protein HSP 90-alpha	P07901		2		Y	BioGRID
Hspa1a	Heat shock 70 kDa protein 1A	Q61696	7			Y	BioGRID
Hspa5	78 kDa glucose-regulated protein	P20029	6	5		Y	BioGRID
Hspa8	Heat shock cognate 71 kDa protein	P63017	12	10	5	Y	BioGRID
Hspb1	Heat shock protein beta-1	P14602	2	1		Y	BioGRID
Ide	Insulin-degrading enzyme	Q9JHR7	3				
Ido1	Indoleamine 2,3-Dioxygenase 1	D3YXV1	1				
Ighg1	Ig gamma-1 chain C region secreted form	A0A075B5		3	1		
Jup	Junction plakoglobin	Q02257	29	31	8		
Kif18a	Kinesin-like protein KIF18A	Q91WD7	1				
Lmna	Prelamin-A/C	P48678	2	2	1		
Lyz1	Lysozyme C-1	P17897	2	1	2		
Mbp	Myelin basic protein	P04370-4			3		
Mdh2	Malate dehydrogenase, mitochondrial	P08249	1	1			
Mrpl43	39S ribosomal protein L43, mitochondrial	Q5RL20		1			
Mtap	S-methyl-5-thioadenosine phosphorylase	Q9CQ65	1				
Myh9	Myosin-9	Q8VDD5	4	1		Y	BioGRID
Nagk	N-acetyl-D-glucosamine kinase	Q9QZ08	1				
Nccrp1	F-box only protein 50	A0A0A0MQ	2				
Nme2	Nucleoside diphosphate kinase B	Q01768	3				
Npepps	Puromycin-sensitive aminopeptidase	Q11011	1			Y	BioGRID
Nsf	Vesicle-fusing ATPase	P46460		1			
Pag1	Phosphoprotein associated with	Q3U1F9	1				
Paics	Multifunctional protein ADE2	Q9DCL9		1			
Pcbp1	Poly(rC)-binding protein 1	P60335	1				
Pcdh15	Protocadherin-15	Q99PJ1		7	2		
Pcdhb2	Protocadherin Beta 2	Q91Y00	1				
Pclo	Protein piccolo	Q9QYX7		1			
Pgk1	Phosphoglycerate kinase 1;	P09411	1				
Phgdh	D-3-phosphoglycerate dehydrogenase	Q61753	2			Y，pubmed	Affinity Capture-MS
Pip4k2b	Phosphatidylinositol 5-phosphate 4-kinase	Q80XI4		2			
Pip4k2c	Phosphatidylinositol 5-phosphate 4-kinase	Q91XU3	3	7	8		
Pkm	Pyruvate kinase PKM	P52480	8	17	2		
Pkp1	Plakophilin-1	P97350	11	7	4		
Pkp3	Plakophilin-3	Q9QY23	1	1			
Pkp4	Plakophilin-4	Q68FH0		2	4		
Plcxd3	PI-PLC X domain-containing protein 3	Q8BLJ3			1		
Plec	Plectin	Q9QXS1	9				
Plp1	Myelin proteolipid protein	P60202			1		
Pnp	Purine nucleoside phosphorylase	P23492	1	1			

Pof1b	Protein POF1B	Q8K4L4	2	2			
Prdx1	Peroxiredoxin-1	P35700	3	3		Y	BioGRID
Prdx2	Peroxiredoxin-2	Q61171	1	1	1	Y	BioGRID
Prss1	Serine Protease 1	Q9Z1R9	1				
Psma1	Proteasome subunit alpha type-1	Q9R1P4	1			Y	BioGRID
Psma2	Proteasome subunit alpha type-2	P49722		3		Y	BioGRID
Psma4	Proteasome subunit alpha type-	Q9R1P0		1			
Psma5	Proteasome subunit alpha type-5	Q9Z2U1		1			
Psma6	Proteasome subunit alpha type-6	Q9QUM9	2	2			
Psma7	Proteasome subunit alpha type-7	Q9Z2U0	2	3		Y	BioGRID
Psmb1	Proteasome subunit beta type-1	O09061	2	2		Y	BioGRID
Psmb2	Proteasome subunit beta type-2	Q9R1P3		2		Y	BioGRID
Psmb3	Proteasome subunit beta type-3	Q9R1P1	2	3			
Psmb5	Proteasome subunit beta type-5	O55234	1	2			
Psmb6	Proteasome subunit beta type-6	Q60692	2	1			
Psme3	Proteasome activator complex subunit 3	P61290		3	2		
Ptbp1	Polypyrimidine tract-binding protein 1	P17225	1				
Rab14	Ras-related protein Rab-14	Q91V41	1	1			
Rab3a	Ras-related protein Rab-3A	P63011		1			
Rab6b	Ras-related protein Rab-6B	P61294	1	1			
Rab7a	Ras-related protein Rab-7a	P51150	3				
Rbm6	RNA Binding Motif Protein 6	A0A0A6YY	1				
Rho	Rhodopsin	P15409		4	3		
Rlbp1	Retinaldehyde-binding protein 1	Q9Z275		11	6		
Rnf222	RING finger protein 222	Q8CEF8	1				
Rpsa	40S ribosomal protein SA	P14206	4	2			
Rs1	Retinoschisin	B1AU64		1	1		
Rtcb	tRNA-splicing ligase RtcB homolog	Q99LF4	1				
S100a11	Protein S100-A11	P50543	2				
S100a14	Protein S100-A14	Q9D2Q8		1	1		
Sdr9c7	Short-chain dehydrogenase/reductase	Q8K3P0		2			
Serpinb5	Serpin B5	P70124	3				
Sfn	14-3-3 protein sigma	O70456	2	4			
Slc16a1	Monocarboxylate transporter 1	P53986		1	1		
Slc25a3	Phosphate carrier protein, mitochondrial	Q8VEM8		1	1	Y	BioGRID
Slc25a4	ADP/ATP translocase 1	P48962		9	2		
Slc25a5	ADP/ATP translocase 2;ADP/ATP	P51881		6		Y， pubmed	
Slc26a10	Solute carrier family 26 member 10	F8WGV3		1		Y	BioGRID
Slco2a1	Solute carrier organic anion transporter	Q9EPT5	1				
Slmo2	Protein slowmo homolog 2	Q9CYY7	1				
Smarca5	SWI/SNF-related matrix-associated actin-	Q91ZW3	2			Y	BioGRID
Srgap3	SLIT-ROBO Rho GTPase-activating	Q812A2		9	12		
Sspo	SCO-spondin	Q8CG65			1		
Ssr4	Translocon-associated protein subunit	Q62186	1				
Stxbp1	Syntaxin-binding protein 1	O08599		1			
Tada2b	Transcriptional Adaptor 2B	D3Z4Z0	1	1			
Tbl3	Transducin beta-like protein 3	Q8C4J7			1		
Tbx2	T-box transcription factor TBX2	Q60707	1				
Tfg	Trafficking From ER To Golgi Regulator	Q9Z1A1	6	11	8		
Tgm1	Protein-glutamine gamma-	Q9JLF6	4	5			
Tgm3	Protein-glutamine gamma-	Q08189	1	1	1		
Tmed9	Transmembrane emp24 domain-	Q99KF1	1				
Tpi1	Triosephosphate isomerase	P17751	2	2			
Tpm3	Tropomyosin alpha-3 chain	E9Q7Q3	4			Y	BioGRID
Tuba1b	Tubulin alpha-1B chain	P05213	3	12	3	Y	BioGRID
Tuba1c	Tubulin alpha-1C chain	P68373		12			
Tubb2a	Tubulin beta-2A chain	Q7TMM9		15	5	Y	BioGRID
Tubb3	Tubulin beta-3 chain	Q9ERD7		10		Y	BioGRID
Tubb4a	Tubulin beta-4A chain	Q9D6F9		12		Y	BioGRID
Tubb4b	Tubulin beta-4B chain	P68372		15	6	Y	BioGRID
Tubb5	Tubulin beta-5 chain	P99024	6	13	6		
Txn	Thioredoxin	P10639		1			
Ubc	Polyubiquitin-C	P0CG50	2	3	1	Y	BioGRID
Upp2	Uridine phosphorylase 2;	Q8CGR7	1				
Vcl	Vinculin	Q64727		1			
Vim	Vimentin	P20152		5	2		
Vps26a	Vacuolar protein sorting-associated	P40336	6	12	8		
Vps26b	Vacuolar protein sorting-associated	Q8C0E2	7	18	13		
Vps29	Vacuolar protein sorting-associated	Q9QZ88		6	6		
Vps35	Vacuolar protein sorting-associated	Q9EQH3	26	46	24		
Wdr12	Ribosome biogenesis protein WDR12	Q9JJA4	1				
Yod1	Ubiquitin thioesterase OTU1	Q8CB27	1				
Ywhae	14-3-3 protein epsilon	P62259		4		Y	BioGRID
Ywhaz	14-3-3 protein zeta/delta	P63101	3	3			
Zscan4d	Zinc finger and SCAN domain containing	A7KBS4			1		

Supplementary Table 2. The -Log10 of P-values from one-sided Fisher's exact test and adjusted p-value with Benjamini correction are shown for the KEGG pathway enriched by the VPS35 interacting protein dataset ($P < 0.05$) by DAVID method. Source data are provided as a Source Data file.

KEGG PATHWAY	Count	%	P-Value	Beniamini
Parkinson disease	23	11.7	2.50E-12	3.20E-10
Prion disease	23	11.7	3.30E-12	3.20E-10
Proteasome	12	6.1	1.40E-11	8.50E-10
Huntington disease	22	11.2	2.50E-10	1.20E-08
Amyotrophic lateral sclerosis	23	11.7	1.70E-09	6.30E-08
Alzheimer disease	23	11.7	3.40E-09	1.10E-07
Pathways of neurodegeneration - multiple diseases	25	12.7	6.40E-09	1.70E-07
Motor proteins	16	8.1	3.20E-08	7.60E-07
Spinocerebellar ataxia	13	6.6	2.70E-07	5.60E-06
Salmonella infection	16	8.1	9.10E-07	1.70E-05
Biosynthesis of amino acids	9	4.6	7.90E-06	1.40E-04
Carbon metabolism	9	4.6	1.70E-04	2.70E-03
Gapjunction	7	3.6	8.70E-04	1.30E-02
Glycolysis / Gluconeogenesis.	6	3	1.70E-03	2.40E-02
Phagosome	9	4.6	2.50E-03	3.20E-02
Amoebiasis	7	3.6	2.70E-03	3.20E-02
Endocytosis	11	5.6	2.80E-03	3.20E-02
Regulation of actin cytoskeleton	10	5.1	3.10E-03	3.30E-02
Tight junction	8	4.1	6.10E-03	6.10E-02
Protein processing in endoplasmic reticulum	8	4.1	7.80E-03	7.40E-02
Gastric acid secretion	5	2.5	1.60E-02	1.40E-01
Focal adhesion	8	4.1	1.70E-02	1.40E-01
Arrhythmogenic right ventricular cardiomyopathy.	5	2.5	1.80E-02	1.50E-01
Antigen processing_and presentation	5	2.5	3.00E-02	2.30E-01
Estrogen signaling_pathway.	6	3	3.10E-02	2.30E-01
Apoptosis	6	3	3.20E-02	2.30E-01
Cysteine and methionine metabolism	4	2	3.50E-02	2.40E-01
Legionellosis	4	2	4.50E-02	3.00E-01
Longevity regulating_pathway - multiple species	4	2	4.70E-02	3.00E-01
Phototransduction	3	1.5	4.80E-02	3.00E-01
Proteoglycans in cancer	7	3.6	5.10E-02	3.10E-01
Glyoxylate and dicarboxylate metabolism	3	1.5	6.50E-02	3.80E-01
Leukocyte transendothelial migration	5	2.5	6.80E-02	3.90E-01
Bacterial invasion of epithelial cells	4	2	7.60E-02	4.20E-01
Synaptic vesicle cycle	4	2	7.90E-02	4.20E-01
Platelet activation	5	2.5	8.00E-02	4.20E-01

Supplementary Table 3. One-sided Fisher's test identified GO pathways enriched by the VPS35 interacting protein dataset (Bonferroni adjusted P< 0.05). Source data are provided as a Source Data file.

pathway	target.select	pathway.gn.cnt	odds.ratio	p.value
GOMF_CADHERIN_BINDING	32	310	13.429897	3.628361E-23
GOMF_CELL_ADHESION_MOLECULE_BINDING	37	515	9.239785	3.241902E-21
GOMF_STRUCTURAL_MOLECULE_ACTIVITY	38	690	6.934961	7.992204E-18
GOCC_PROTEASOME_CORE_COMPLEX	11	20	125.808524	1.375299E-17
GOMF_STRUCTURAL_CONSTITUENT_OF_CYTOSKELETON	16	107	18.678653	1.296076E-14
GOCC_VESICLE_LUMEN	24	320	8.956110	2.141393E-14
GOCC_PROTEASOME_COMPLEX	13	59	29.529242	2.170211E-14
GOCC_SUPRAMOLECULAR_COMPLEX	46	1310	4.424669	4.856519E-14
GOCC_SUPRAMOLECULAR_POLYMER	39	972	4.926290	9.941666E-14
GOCC_CELL_SUBSTRATE_JUNCTION	26	425	7.248205	1.965913E-13
GOCC_FICOLIN_1_RICH_GRANULE	18	183	11.689584	4.681526E-13
GOCC_ANCHORING_JUNCTION	35	866	4.854146	1.881407E-12
GOCC_FICOLIN_1_RICH_GRANULE_LUMEN	15	124	14.519951	2.178910E-12
GOCC_SECRETORY_GRANULE	34	851	4.766592	5.656036E-12
GOCC_ENDOPEPTIDASE_COMPLEX	13	89	17.861998	5.788543E-12
GOCC_ACTIN_CYTOSKELETON	25	468	6.221565	1.170049E-11
GOCC_CORNIFIED_ENVELOPE	11	58	24.169918	1.334849E-11
GOCC_PEPTIDASE_COMPLEX	14	122	13.597438	2.508289E-11
GOCC_PROTEASOME_CORE_COMPLEX_ALPHA_SUBUNIT_COMPLEX	6	8	300.152206	2.808863E-11
GOCC_SECRETORY_VESICLE	36	1009	4.257427	3.102689E-11
GOMF_PROTEIN_CONTAINING_COMPLEX_BINDING	39	1209	3.870757	8.187922E-11
GOBP_CYTOSKELETON_ORGANIZATION	41	1413	3.477183	6.084868E-10
HP_CONGENITAL_ONSET	30	832	4.173019	1.381984E-09
GOMF_CYTOSKELETAL_PROTEIN_BINDING	32	945	3.937260	1.697762E-09
GOCC_CONTRACTILE_FIBER	16	239	7.567977	3.036798E-09
GOBP_ORGANONITROGEN_COMPOUND_CATABOLIC_PROCESS	38	1320	3.394153	3.727983E-09
GOCC_DESMOSOME	7	27	35.351267	7.505602E-09
GOMF_ACTIN_FILAMENT_BINDING	14	200	7.858883	1.740729E-08
GOCC_MYELIN_SHEATH	8	46	21.361861	1.841987E-08
GOMF_RNA_BINDING	44	1802	2.905428	2.521023E-08
GOCC_ADHERENS_JUNCTION	13	175	8.339691	2.834365E-08
GOCC_CELL_CORTEX	16	282	6.328512	3.178843E-08
HP_NEONATAL_DEATH	9	70	15.055249	3.643916E-08
GOMF_GUANYL_NUCLEOTIDE_BINDING	18	366	5.484353	3.823958E-08
GOCC_PROTEASOME_CORE_COMPLEX_BETA_SUBUNIT_COMPLEX	5	11	83.166202	4.534213E-08
GOBP_HOMOTYPIC_CELL_CELL_ADHESION	10	97	11.781820	5.410779E-08
GOMF_ACTIN_BINDING	19	424	4.988070	6.856998E-08
GOCC_POLYMERIC_CYTOSKELETAL_FIBER	25	723	3.889639	8.578181E-08
GOBP_SUPRAMOLECULAR_FIBER_ORGANIZATION	26	777	3.772632	8.796647E-08
GOMF_STRUCTURAL_CONSTITUENT_OF_EYE_LENS	6	23	35.446973	8.920227E-08
GOBP_MAINTENANCE_OF_PRESYNAPTIC_ACTIVE_ZONE_STRUCTURE	4	6	198.104774	1.530337E-07
GOCC_PIGMENT_GRANULE	10	112	10.038218	2.135279E-07
GOMF_PURINE_NUCLEOTIDE_BINDING	43	1877	2.688434	2.326810E-07
GOBP_CELL_JUNCTION_ORGANIZATION	24	723	3.703760	3.361401E-07
GOCC_PRESYNAPTIC_ACTIVE_ZONE_CYTOPLASMIC_COMPONENT	5	17	41.620313	5.779297E-07
GOBP_PRESYNAPTIC_ACTIVE_ZONE_ORGANIZATION	4	8	99.211308	7.028421E-07
HP_PLANTAR_HYPERKERATOSIS	11	161	7.535401	7.896227E-07
HP_ABNORMALITY_OF_THE_PLANTAR_SKIN_OF_FOOT	12	196	6.727870	7.979008E-07
GOBP_PLATELET_AGGREGATION	8	74	12.291787	8.350334E-07
GOCC_CORTICAL_CYTOSKELETON	9	102	9.859606	9.712035E-07
GOBP_INTRACELLULAR_TRANSPORT	36	1502	2.740564	9.925140E-07
HP_INABILITY_TO_WALK	15	321	5.113908	1.046220E-06
HP_FRAGILE_SKIN	6	34	21.529040	1.083039E-06
GOBP_CELL_CELL_JUNCTION_ORGANIZATION	12	208	6.312063	1.494796E-06
HP_RESPIRATORY_INSUFFICIENCY	20	574	3.829357	1.666601E-06
GOBP_ESTABLISHMENT_OF_PROTEIN_LOCALIZATION	37	1604	2.636966	1.701453E-06
HP_MYOPATHY	14	291	5.248932	1.724948E-06
GOCC_SYNAPSE	34	1412	2.730422	1.906420E-06
GOCC_CELL_CORTEX_REGION	6	38	18.837000	2.149407E-06
GOBP_CELL_CELL_ADHESION	26	924	3.126786	2.372021E-06
GOCC_CELL_CELL_JUNCTION	18	487	4.040281	2.508110E-06
GOBP_PROTEIN_REFOLDING	5	23	27.728190	2.990796E-06
GOMF_CELL_ADHESION_MEDIATOR_ACTIVITY	7	62	12.838722	3.080051E-06

Supplementary Table 4. GSEA method identified GO pathways enriched by the VPS35 interacting protein dataset (FDR < 0.2). Source data are provided as a Source Data file.

NAME	SIZE	ES	NES	NOM p-val	FDR q-val
GOMF_STRUCTURAL_CONSTITUENT_OF_CYTOSKELETON	15	0.678821	3.182758	0.000000	0.000000
GOCC_POLYMERIC_CYTOSKELETAL_FIBER	26	0.525792	2.995497	0.000000	0.000000
GOCC_SUPRAMOLECULAR_POLYMER	40	0.394231	2.706170	0.000000	0.002874
GOBP_CYTOSKELETON_ORGANIZATION	42	0.303030	2.103099	0.002105	0.160078
GOBP_REGULATION_OF_CATABOLIC_PROCESS	23	0.384267	2.097894	0.001890	0.133266
GOCC_MICROTUBULE_CYTOSKELETON	29	0.349783	2.084053	0.002033	0.117236
GOBP_NEGATIVE_REGULATION_OF_MOLECULAR_FUNCTION	18	0.426966	2.072460	0.002028	0.105736
GOCC_SUPRAMOLECULAR_COMPLEX	46	0.289275	2.056459	0.001912	0.100695
GOBP_POSITIVE_REGULATION_OF_MOLECULAR_FUNCTION	15	0.441989	2.009602	0.002016	0.117993
GOBP_MICROTUBULE_BASED_PROCESS	24	0.364341	2.003995	0.008282	0.109742
GOMF_IDENTICAL_PROTEIN_BINDING	55	0.272083	1.997589	0.007813	0.104540
GOMF_STRUCTURAL_MOLECULE_ACTIVITY	37	0.305966	1.991005	0.009960	0.100823
GOBP_RESPONSE_TO_ENDOGENOUS_STIMULUS	27	0.340127	1.983023	0.006110	0.097986
GOBP_MICROTUBULE_CYTOSKELETON_ORGANIZATION	20	0.393182	1.973746	0.001969	0.095154
GOMF_HYDROLASE_ACTIVITY_ACTING_ON_ACID_ANHYDRIDES	22	0.362069	1.952090	0.004082	0.103626
GOCC_ANCHORING_JUNCTION	35	0.294410	1.941019	0.008065	0.103850
GOMF_RIBONUCLEOTIDE_BINDING	39	0.289237	1.938565	0.009728	0.099569
GOBP_CELL_CYCLE	34	0.304285	1.909142	0.008197	0.110802
GOBP_POSITIVE_REGULATION_OF_GENE_EXPRESSION	24	0.345930	1.908624	0.007952	0.105341
GOCC_CELL_CELL_JUNCTION	18	0.384519	1.894210	0.007533	0.107615
GOBP_INTRACELLULAR_TRANSPORT	36	0.288889	1.884297	0.004000	0.109079
GOBP_CELL_JUNCTION_ORGANIZATION	23	0.348077	1.881970	0.013462	0.105742
GOBP_CELLULAR_RESPONSE_TO_OXYGEN_CONTAINING_COMPOUND	23	0.349334	1.879042	0.013972	0.102758
GOBP_RESPONSE_TO_ABIOTIC_STIMULUS	22	0.350052	1.870839	0.012195	0.102625
GOMF_GUANYL_NUCLEOTIDE_BINDING	17	0.384160	1.849778	0.011342	0.110105
GOBP_NEGATIVE_REGULATION_OF_RESPONSE_TO_STIMULUS	19	0.360095	1.823886	0.009690	0.121263
GOCC_VESICLE_MEMBRANE	20	0.355682	1.804972	0.011881	0.127475
GOBP_MITOTIC_CELL_CYCLE	16	0.381944	1.784020	0.012024	0.138639
GOBP_RESPONSE_TO_NITROGEN_COMPOUND	23	0.323448	1.773975	0.017341	0.142223
GOBP_TISSUE_DEVELOPMENT	30	0.284739	1.758102	0.022514	0.149341
GOBP_DEFENSE_RESPONSE_TO_OTHER_ORGANISM	17	0.359185	1.755084	0.024194	0.147320
GOCC_CELL_SUBSTRATE_JUNCTION	26	0.299095	1.748223	0.023121	0.147822
GOBP_RESPONSE_TO_CYTOKINE	19	0.336010	1.713156	0.020873	0.171853
GOBP_CELLULAR_RESPONSE_TO_NITROGEN_COMPOUND	17	0.360171	1.712612	0.027833	0.167108
GOBP_CELLULAR_RESPONSE_TO_ENDOGENOUS_STIMULUS	22	0.311390	1.704733	0.028226	0.168798
GOBP_REGULATION_OF_CELL_DEATH	30	0.285141	1.696224	0.024668	0.171918
HP_FUNCTIONAL_ABNORMALITY_OF_THE_INNER_EAR	20	0.337500	1.689714	0.036735	0.173019
GOBP_RESPONSE_TO_HORMONE	16	0.362500	1.689171	0.033543	0.168842
GOBP_NEGATIVE_REGULATION_OF_SIGNALING	15	0.363536	1.669853	0.026000	0.182220
GOCC_MEMBRANE_PROTEIN_COMPLEX	16	0.355556	1.646917	0.026477	0.198266
GOBP_PROGRAMMED_CELL_DEATH	35	0.253416	1.642533	0.048170	0.196649

Supplementary Table 5: **List of antibodies and dyes** used this paper. Ab: antibody; IB: immunoblotting; IF: immunofluorescence staining.

Target	Description	Host	Sources	Clone and/or Cat#	Ab registry/validation	Assays/Dilution
ABCA4	1° Ab, non-conjugated	Rabbit	Hui Sun		[2]	IB 1:2,000
ATP1A	1° Ab, non-conjugated	Mouse	DSHB	a5 sup	AB_2166869	IB: 1:150
CD63	1° Ab, non-conjugated	Rat	Biolegend	143901	AB_11203908	IF 1:200
CD68	1° Ab, Violet 605 conjugated	Rat	Biolegend	137021	AB_2616811	IF 1:100
Cre	1° Ab, non-conjugated	Mouse	Millipore	MAB3120	AB_208574	IF 1: 500
Cone arrestin	1° Ab, non-conjugated	Rabbit	Millipore	AB15282	AB_1163387	IF: 1,1000
EEA1	1° Ab, non-conjugated	Mouse	BD Biosciences	610456	AB_397829	IF 1:500
EEA1	1° Ab, non-conjugated	Goat	SCBT	sc-6414	AB_640035	IF 1:100
GAPDH	1° Ab, non-conjugated	Rabbit	CST	2118	AB_561053	IB 1:5,000
Glutamine synthase	1° Ab, non-conjugated	Mouse	BD Biosciences	610517	AB_397879	IF 1:4,000
HSC70	1° Ab, non-conjugated	Rabbit	Proteintech	10654-1-AP	AB_2120153	IF 1:200<br>IB: 1:3,000
Iba1	1° Ab, non-conjugated	Rabbit	Wako	019-19741	AB_839504	IF 1:500
IL1β	1° Ab, non-conjugated	Mouse	CST	12242	AB_2715503	IF: 1:100
IRBP	1° Ab, non-conjugated	Rabbit	Shao-Ling Fong		[3]	IF 1:100<br>IB 1:2,500
Lamp1	1° Ab, non-conjugated	Rat	DSHB	1D4B	AB_2134500	IF 1:400
LC3A/B-I/II	1° Ab, non-conjugated	Rabbit	Cell Signaling Technology	4108	AB_2137703	IF 1:200<br>IB 1:1,000
LC3A/B-I/II	1° Ab, non-conjugated	Mouse	MBL	M152-3	AB_1279144	IF 1:500
mGluR6	1° Ab, non-conjugated	Sheep	Jeannie Chen		[4]	IF 1:5,000
Myc	1° Ab, non-conjugated	Mouse	SCBT	sc-40	AB_627268	IF 1:200

PDE6	1° Ab, non-conjugated	Rabbit	Rehwa H. Lee	pat-B	[5]	IB: 1:2,000
Peripherin 2/rds	1° Ab, non-conjugated	Mouse	Robert S. Molday	Per 5H2	[6]	IF 1:15
Peripherin 2/rds	1° Ab, non-conjugated	Rabbit	Novusbio	NBP1-86687	AB_11006045	IB 1:1,000
Rhodopsin	1° Ab, Alexa488 conjugated	Mouse	Homemade	B6-30	[7]	IF 1:5,000
Rhodopsin	1° Ab, non-conjugated	Mouse	Paul A. Hargrave	B6-30	[7]	IB 1:3,000
Ribeye	1° Ab, non-conjugated	Rabbit	Frank Schmitz	N-and C-terminus	[8]	IF 1:100
Snap25	1° Ab, non-conjugated	Mouse	Covance	SMI-81R	AB_510034	IF 1:4000
αSyn	1° Ab, non-conjugated	Mouse	BD biosience	610786	AB_398107	IF: 1:500<br>IB: 1,000
Phsopho(S129)αSyn	1° Ab, non-conjugated	Rabbit	Abcam	AB51253	AB_869973	IF: 1:1,000<br>IB: 1,3,000
α-Tubulin	1° Ab, non-conjugated	Mouse	Millipore	05-829 (DM1A)	AB_310035	IB 1:5000
VPS26	1° Ab, non-conjugated	Rabbit	Abcam	Ab23892	AB_2215043	IB: 1,1000
VPS35	1° Ab, non-conjugated	Goat	Novusbio	NB100-1397	AB_2257186	IF 1:200
Ubiquitin	1° Ab, non-conjugated	Mouse	Santa Cruz	sc-8017	AB_628423	IF: 1:50
Goat IgG	2° Ab, Alexa488 conjugated	Donkey	ThermoFisher	A11055	AB_2534102	IF 1:400
Goat IgG	2° Ab, Alexa568 conjugated	Donkey	ThermoFisher	A11057	AB_2534104	IF 1:400
Goat IgG	2° Ab, Alexa647 conjugated	Donkey	ThermoFisher	A-21447	AB_2535864	IF 1:400
Mouse IgG	2° Ab, Alexa568 conjugated	Donkey	ThermoFisher	A10037	AB_2534013	IF 1:400
Mouse IgG	2° Ab, Alexa647 conjugated	Donkey	ThermoFisher	A-31571	AB_162542	IF 1:400
Mouse IgG	2° Ab, IRDye680 conjugated	Donkey	LI-COR	926-32222	AB_621844	IB 1:10,000
Mouse IgG	2° Ab, IRDye800 conjugated	Goat	LI-COR	926-32210	AB_621842	IB 1:10,000
Rabbit IgG	2° Ab, Alexa488 conjugated	Donkey	Thermo Fisher	A21206	AB_2535792	IF 1:400

Rabbit IgG	2° Ab, Alexa568 conjugated	Donkey	ThermoFisher	A10042	AB_2534017	IF 1:400
Rabbit IgG	2° Ab, IRDye680 conjugated	Donkey	LI-COR	926-32223	AB_621845	IB 1:10,000
Rabbit IgG	2° Ab, IRD800CW conjugated	Donkey	LI-COR	926-32213	AB_621848	IB 1:10,000
Rat IgG	2° Ab, Alexa488 conjugated	Donkey	ThermoFisher	A-21208	AB_2535794	IF 1:400
Rat IgG	2° Ab, Alexa647 conjugated	Donkey	Jackson ImmunoResearch Inc.	712-605-153	AB_2340694	IF 1:400
Rat IgG	2° Ab, IRDye 800CW conjugated	Goat	LI-COR	926-32219	AB_1850025	IB 1:10,000
Rat IgG	2° antibody, Alexa594 conjugated	Donkey	Jackson ImmunoResearch Labs	712-585-153	AB_2340689	IF 1:400
Goat IgG	2° Ab, IRDye 800CW conjugated	Donkey	LI-COR	926-32214	AB_621846	IB 1:10,000
Revert™ 700 Total Protein Stain		LI-COR	926-11015			Revert™ 700 Total Protein Stain

Supplementary Table 6: List of primers used for genotyping and qPCR.

Gene ID		Primer	Assay used
iCre	Forward	5'-CCACCTCTGATGAAGTCAGGA-3'	Genotyping (iCre, 311 bp)
	Reverse	5'-TCTGATTCTCCTCATCACCAG-3'	
Vps35	Forward	5'-AACCAGCTCCCAACAAAATG-3'	Genotyping (WT, 155 bp; Floxed, 220 bp)
	Reverse	5'-AAATGTGAGTGGGACCAAGC-3'	
Rpe65	Forward	5'-TGAGTCATATGCTAGTCAAGT-3'	Genotyping Rpe65Leu450Met
	Reverse	5'-ATCTTCTTCCAGAGCATCTGGTTG-3'	
Crb1	Forward1	5'-GTGAAGACAGCTACAGTTCTGATC-3'	Genotyping (WT, 220 bp; rd8, 244bp)
	Forward2	5'-GCCCCTGTTTGCATGGAGGAAACTT GGAAGACAGCTACAGTTCTTCTG-3'	
	Reverse	5'-GCCCCATTTGCACACTGATGAC-3'	
Abca4	Forward	5’-AACACCTTTACCTCTATGCCAG-3'	qPCR
	Reverse	5’-TTTCCTCTTATTGCCTCCACTG-3'	
Gapdh	Forward	5'-CCTTCCGTGTTCCTACCC-3'	qPCR
	Reverse	5'-CAACCTGGTCCTCAGTGTAG-3'	
Irbp	Forward	5’-CTGGACAAGATCTACAACCGG-3'	qPCR
	Reverse	5’-CAGGATGGCTACGCTCTTC -3'	
Prph2	Forward	5’-CAATCGCTACCTGGACTTCTC-3'	qPCR
	Reverse	5’-GTTGGTGAGCTGGTACTGG-3'	
	Reverse	5’-ATCTCTGACAATACAACGCCC	
Rho	Forward	5'-CCCTTCTCCAACGTCACAGG-3'	qPCR
	Reverse	5'-TGAGGAAGTTGATGGGGAAGC-3'	

Supplementary Table 7: List of plasmids used in this paper.

Plasmid	Source
pRK5	Gift of Jeremy Nathans [9]
Scramble shRNA	Targeting sequences CTCATTCCTCGTGGTCCGTCAT
VPS35-shRNA1	Targeting sequences GGGTGCGGATGCAGCATCAAGG
VPS35-shRNA 2	Targeting sequences ACCAGGTAGATTCCATAATGAA
CMV: Flag-Rab5Q79L	Homemade [10]
CMV: Myc-(human wildtype) αSyn	Gift from Jacqueline Burre[11]
mCherry-(mouse)VPS35	Gift from Mark von Zastrow [12]
GST-(mouse) VPS35 Full-length (M1-L796)	Homemade
GST-(mouse) VPS35 C-terminal motif (L302-L796)	Homemade
GST-(mouse) VPS35 N-terminal motif (M1-A301)	Homemade
His-HSPA8 (HSC70)	Addgene 177661[13]

Supplemental references

1. Solovei I*, et al.* Nuclear architecture of rod photoreceptor cells adapts to vision in mammalian evolution. *Cell* **137**, 356-368 (2009).

2. Sun H, Nathans J. Stargardt's ABCR is localized to the disc membrane of retinal rod outer segments. *Nat Genet* **17**, 15-16 (1997).

3. Bridges CD, Fong SL, Landers RA, Liou GI, Font RL. Interstitial retinol-binding protein (IRBP) in retinoblastoma. *Neurochem Int* **7**, 875-881 (1985).

4. Cao Y*, et al.* Retina-specific GTPase accelerator RGS11/G beta 5S/R9AP is a constitutive heterotrimer selectively targeted to mGluR6 in ON-bipolar neurons. *J Neurosci* **29**, 9301-9313 (2009).

5. Suber ML*, et al.* Irish setter dogs affected with rod/cone dysplasia contain a nonsense mutation in the rod cGMP phosphodiesterase beta-subunit gene. *Proc Natl Acad Sci U S A* **90**, 3968-3972 (1993).

6. Connell G, B□ascom R, Molday L, Reid D, McInnes R, Molday RS. Photoreceptor peripherin is the normal product of the gene responsible for retinal degeneration in the rds mouse. *Proc Natl Acad Sci USA* **88**, 723-726 (1991).

7. Adamus G, Arendt A, Hargrave PA. Genetic control of antibody response to bovine rhodopsin in mice: epitope mapping of rhodopsin structure. *J Neuroimmunol* **34**, 89-97 (1991).

8. Schmitz F, Konigstorfer A, Sudhof TC. RIBEYE, a component of synaptic ribbons: a protein's journey through evolution provides insight into synaptic ribbon function. *Neuron* **28**, 857-872 (2000).

9. Sung C-H, Davenport CM, Nathans J. Rhodopsin mutations responsible for autosomal dominant retinitis pigmentosa: clustering of functional classes along the polypeptide chain. *J Biol Chem* **268**, 26645-26649 (1993).

10. Hsu KS*, et al.* CLIC4 regulates late endosomal trafficking and matrix degradation activity of MMP14 at focal adhesions in RPE cells. *Sci Rep* **9**, 12247 (2019).

11. Burre J, Sharma M, Sudhof TC. Systematic mutagenesis of alpha-synuclein reveals distinct sequence requirements for physiological and pathological activities. *J Neurosci* **32**, 15227-15242 (2012).

12. Choy RW, Park M, Temkin P, Herring BE, Marley A, Nicoll RA, von Zastrow M. Retromer mediates a discrete route of local membrane delivery to dendrites. *Neuron* **82**, 55-62 (2014).

13. Ayoub CA, Wagner CS, Kuret J. Identification of gene networks mediating regional resistance to tauopathy in late-onset Alzheimer's disease. *PLoS Genet* **19**, e1010681 (2023).
