# Supplementary material for: Mutant mice with rod-specific VPS35 deletion exhibit retinal α-synuclein pathology-associated degeneration
Source: Nat Commun. 2024 Jul 23;15:5970. doi: 10.1038/s41467-024-50189-0 (PMC11266608; doi:10.1038/s41467-024-50189-0)
Supplement: Supplementary file 3 — Description of Additional Supplementary Files [file 41467_2024_50189_MOESM3_ESM.docx]

**Description of Additional Supplementary Files**

**Supplementary Movie 1:**

Support of Figs. 4e and 4f. A stack of FIB-SEM images (z-step = 10 nm) of two rod terminals (Rt1, Rt2) of 3-monthold KO that are filled up with various membrane debris. Most notably, MLBs and LEs whose swollen lumen contains either “clear” vesicles or fuzzy aggregates. LEs are subsequently deposited as intracellular lipofuscins, which are eventually expelled into the extracellular space along with other membranous debris.

**Supplementary Movie 2:**

An image stack (z-step= 10 nm) of a rod terminal of 3-month-old KO shows the heterogenous-looking LEs in which the lumens contain SVs and amorphous vesicular aggregates (arrowheads). The coalescence of the LE luminal aggregates forms future lipofuscins that are deposited into cytosols from permeable LE-limiting membranes.

**Supplementary Movie 3:**

Support of Fig. 4b. An image stack (z-step= 10 nm) of the IS of a rod of 3-month-old KO shows a constellation of LE vacuoles with heterogenous luminal contents and the lipofuscin (Lf) deposited nearby.

**Supplementary Movie 4:**

Support of Fig. 4c. An image stack (z-step= 40 nm) shows similar profiles of lipofuscins and their precursors (i.e., amorphous vesicular aggregates) are abundant in rod terminals (shaded in red) and microglia (shaded in blue) of 3-monthold KO, albeit the lipofuscins in microglia expressed tend to have a higher electron density
