# Supplementary material for: Mutant mice with rod-specific VPS35 deletion exhibit retinal α-synuclein pathology-associated degeneration
Source: Nat Commun. 2024 Jul 23;15:5970. doi: 10.1038/s41467-024-50189-0 (PMC11266608; doi:10.1038/s41467-024-50189-0)
Supplement: Supplementary file 8 — Reporting Summary [file 41467_2024_50189_MOESM8_ESM.pdf]

nature portfolio

Corresponding author(s): Ching-Hwa Sung

Last updated by author(s): June 7, 2024

Reporting Summary

Nature Portfolio wishes to improve the reproducibility of the work that we publish. This form provides structure for consistency and transparency in reporting. For further information on Nature Portfolio policies, see our Editorial Policies and the Editorial Policy Checklist.

Statistics

For all statistical analyses, confirm that the following items are present in the figure legend, table legend, main text, or Methods section.

n/a	Confirmed	
☐	☒	The exact sample size (*n*) for each experimental group/condition, given as a discrete number and unit of measurement
☐	☒	A statement on whether measurements were taken from distinct samples or whether the same sample was measured repeatedly
☐	☒	The statistical test(s) used AND whether they are one- or two-sided<br>*Only common tests should be described solely by name; describe more complex techniques in the Methods section.*
☒	☐	A description of all covariates tested
☒	☐	A description of any assumptions or corrections, such as tests of normality and adjustment for multiple comparisons
☐	☒	A full description of the statistical parameters including central tendency (e.g. means) or other basic estimates (e.g. regression coefficient) AND variation (e.g. standard deviation) or associated estimates of uncertainty (e.g. confidence intervals)
☐	☒	For null hypothesis testing, the test statistic (e.g. *F*, *t*, *r*) with confidence intervals, effect sizes, degrees of freedom and *P* value noted<br>*Give P values as exact values whenever suitable.*
☒	☐	For Bayesian analysis, information on the choice of priors and Markov chain Monte Carlo settings
☒	☐	For hierarchical and complex designs, identification of the appropriate level for tests and full reporting of outcomes
☐	☒	Estimates of effect sizes (e.g. Cohen's *d*, Pearson's *r*), indicating how they were calculated

Our web collection on statistics for biologists contains articles on many of the points above.

Software and code

Policy information about availability of computer code

Data collection	No custom computer code or algorithm was used to generate results. Commercial software used: InSightv2.0.6080, OptoDrum v1.5.0, Espion V6, Spectralis v6.3a, MaxQuant v2.6.1.0, GraphPad Prism V10.2.2 (341), Adobe photoshop 25.9, Adobe illustrator 28.5.<br>Open Access: DAVID (https://david.ncifcrf.gov/tools.jsp), Image J2 (V2.14.0/1.54f (plugins). All softwares used were also described in mauscript.
Data analysis	1) The retinal layers were segmented and measured using the Insight software (InSightv2.0.6080) . Measurement from 400um away from the optic nerve head were used for data analysis ((2) The visual acuity of mice was measured using the OptoDrum (OptoDrum v1.5.0) consisting of an infra-red digital camera which recored their head movements on exposure) to rotating images of black vertical bars at different contrast and rotation speed. (3) ERG measurements were done using the Espion e2 Visual Electrophysiology System (Espion V6). (4) Subretinal layer AF dot counts were determined from images acquired by Heidelberg cSLO (Spectralis v6.3a). (5) For protein identification and quantification, the raw data were processed by MaxQuant (6) The enriched pathways of VPS35-interacting proteins was analyzed with DAVID (one sided Fisher's exact test). One-sided Fisher's test to identify GO pathways (Bonferroni adjusted P< 0.05) and GSEA method to identify GO pathways (FDR < 0.2). (7) Image analysis for expression levels and cell morphology was using Image J (Frac Lac and JACoP plugin).

For manuscripts utilizing custom algorithms or software that are central to the research but not yet described in published literature, software must be made available to editors and reviewers. We strongly encourage code deposition in a community repository (e.g. GitHub). See the Nature Portfolio guidelines for submitting code & software for further information.

Data

Policy information about availability of data

All manuscripts must include a data availability statement. This statement should provide the following information, where applicable:

- Accession codes, unique identifiers, or web links for publicly available datasets
- A description of any restrictions on data availability
- For clinical datasets or third party data, please ensure that the statement adheres to our policy

The authors declare that data supporting the findings of this study are available within the main text and supplementary materials. Source data are provided with this paper, including data of main figures (1d, 1e, 2a, 2b, 2c, 2d, 3c, 3e, 3f, 3g, 5b, 5f, 6h, 7b, 7i, 8c, 8d, 8e, and 8f), Supplementary figures (1d, 1f, 1h,2a, 2c, 3c, 4b, 6c, 6d, 7b, 7c), Pearson's coefficients (related to Figs 3h and 7h) and Supplementary tables 1-4.The proteomics data generated in this study have been deposited in the ProteomicXchange Pride database. Accession: PXD044890. All data are available from the corresponding author (chsung@med.cornell.edu) upon request.

Research involving human participants, their data, or biological material

Policy information about studies with human participants or human data. See also policy information about sex, gender (identity/presentation), and sexual orientation and race, ethnicity and racism.

Reporting on sex and gender	N/A
Reporting on race, ethnicity, or other socially relevant groupings	N/A
Population characteristics	N/A
Recruitment	N/A
Ethics oversight	N/A

Note that full information on the approval of the study protocol must also be provided in the manuscript.

Field-specific reporting

Please select the one below that is the best fit for your research. If you are not sure, read the appropriate sections before making your selection.

☒ Life sciences ☐ Behavioural & social sciences ☐ Ecological, evolutionary & environmental sciences

For a reference copy of the document with all sections, see nature.com/documents/nr-reporting-summary-flat.pdf

Life sciences study design

All studies must disclose on these points even when the disclosure is negative.

Sample size	Preliminary experiments were performed when possible to determine sample size, taking into account resources available and ethical, reductionist animal use.
Data exclusions	All attempts at replication were successful. No data was excluded from the analysis.
Replication	Numbers of the biological and experimental replications were indicated in each figure legends.The number of mice used are described in figure legends.
Randomization	All animals were assigned to groups based on their genotype.
Blinding	The data collection and analyses of mouse experiments and other experiments were blinded. For data collection, the investigators were blinded to the animal's genotypes or transfected please. The investigators were not blinded to group allocation during data collection. However, investigator bias is not considered to contribute to the data because the investigator was blinded at the time of data analysis. Proteomics and pathway analyses were performed by a biostatisticians who were blinded to experimental groups.

Reporting for specific materials, systems and methods

We require information from authors about some types of materials, experimental systems and methods used in many studies. Here, indicate whether each material, system or method listed is relevant to your study. If you are not sure if a list item applies to your research, read the appropriate section before selecting a response.

Materials & experimental systems

n/a	Involved in the study
[ ]	[x] Antibodies
[ ]	[x] Eukaryotic cell lines
[x]	[ ] Palaeontology and archaeology
[ ]	[x] Animals and other organisms
[x]	[ ] Clinical data
[x]	[ ] Dual use research of concern
[x]	[ ] Plants

Methods

n/a	Involved in the study
[x]	[ ] ChIP-seq
[x]	[ ] Flow cytometry
[x]	[ ] MRI-based neuroimaging

Antibodies

Antibodies used

Both primary and secondary antibodies were listed in Supplementary Table 4 and listed below. The detail sources, clones, catlogue numbers, references of validation, and dilution folds used for different assays were also listed.

Primary antibodies
ABCA4 rabbit antibody (gift from Dr. Hui Sun)
ATP1A mouse antibody (DSHB, A5 sup)
CD63 rat antibody（Biolegend, #143901)
CD68 rat antibody （Biolegend, #137021)
Cre mouse antibody (Millipore, #MAB3120)
Cone arrestin rabbit antibody (Millipore, #AB15282)
EEA1 mouse antibody (BD Biosciences, #610456);
EEA1 goat antibody (Santa Cruz, sc-6414)
GAPDH rabbit antibody (cell signaling technology, #2118)
Glutamine synthase mouse antibody (BD Biosciences, #610517)
HSC70 rabbit antibody (Proteintech, #10654-1-AP)
Iba1 rabbit antibody (Wako, #019-19741)
IL1β mouse antibody (cell signaling technology, #12242)
IRBP rabbit antibody (gift from Dr. Shao-Ling Fong)
Lamp1 rat antibody (DSHB, #1D4B)
LC3A/B-I/II rabbit antibody (cell signaling technology, #4108)
LC3A/B-I/II mouse antibody (MBL, #M152-3)
mGluR6 sheep antibody (gift from Dr. Jeannie Chen)
Myc mouse antibody (Santa Cruz, #sc-40)
PDE6 rabbit antibody (gift from Dr. Rehwa H. Lee, clone pat-B)
Peripherin2/rds mouse antibody (gift from Dr. Robert S. Molday, clone Per5H2
Peripherin2/rds rabbit antibody (Novusbio, #NBP1-86687)
Rhodopsin Alexa488 conjugated mouse antibody (Homemade, clone B6-30)
Rhodopsin mouse antibody (gift from Dr. Paul A. Hargrave, clone B6-30)
Ribeye rabbit antibody (gift from Dr. Frank Schmitz, N-and C-terminus)
Snap25 mouse antibody (Covance, #SMI-81R)
αSyn mouse antibody (BD biosciences, #610786)
Phsopho(S129)αSyn rabbit antibody (Abcam, #AB51253)
α-Tubulin mouse antibody (Millipore, #05-829 (DM1A))
VPS26 rabbit antibody (Abcam, #Ab23892)
VPS35 goat antibody (Novusbio, #NB100-1397)
Ubiquitin mouse antibody (Santa Cruz, #sc-8017)

Secondary antibodies:
Alexa488-conjugated donkey anti-goat IgG (ThermoFisher #A11055); Alexa568-conjugated donkey anti-goat IgG (ThermoFisher #A11057); Alexa647-conjugated donkey anti-goat IgG (ThermoFisher #A-21447); Alexa568-conjugated donkey anti-mouse IgG (ThermoFisher #A10037); Alexa647-conjugated donkey anti-mouse IgG (ThermoFisher #A-31571); IRDye680 conjugated donkey anti-mouse IgG (LI-COR #926-32222); IRDye800 conjugated goat anti-mouse IgG (LI-COR #926-32210); Alexa488-conjugated donkey anti-rabbit (Thermo Fisher #A21206); Alexa568-conjugated donkey anti-rabbit IgG (ThermoFisher #A10042); IRDye680 conjugated donkey anti-rabbit IgG (LI-COR #926-32223); IRD800CW conjugated donkey anti-rabbit IgG (LI-COR #926-32213); Alexa488-conjugated donkey anti-rat IgG (ThermoFisher #A-21208); Alexa647-conjugated donkey anti-rat IgG (Jackson ImmunoResearch #712-605-153); IRDye 800CW conjugated goat anti-rat IgG (LI-COR #926-32219); Alexa594 conjugated donkey anti-rat IgG (Jackson ImmunoResearch #712-585-153); IRDye 800CW conjugated donkey-anti goat IgG (LI-COR #926-32214); Revert™ 700 Total Protein Stain (LI-COR #926-11015).

All antibodies listed in Supplemental Table 4

Validation

All the validation is listed in the 6th column "Ab registry/validation" in Supplemental Table 5.

Eukaryotic cell lines

Policy information about cell lines and Sex and Gender in Research

Cell line source(s)	661W was acquired from Muayyad Al-Ubaidi (University of Houston)
Authentication	RRID:CVCL_6240
Mycoplasma contamination	The cell line was tested for mycoplasma infection in donor's lab
Commonly misidentified lines (See ICLAC register)	N/A

Animals and other research organisms

Policy information about studies involving animals; ARRIVE guidelines recommended for reporting animal research, and Sex and Gender in Research

Laboratory animals	The iCre75 mice 40 (from Ching-Kang Chen) and Vps35f/f mice 77 (from Wen-Cheng Xiong) were bred on the background of C57BL6/J (Jackson Lab). We crossed iCre+/-: Vps35f/f males with Vps35f/f females to generate age-matched iCre75+/-; Vps35f/f (KO) and iCre75-/-; Vps35f/f (Ctrl) littermates for experiments. We experimentally confirmed our mouse stocks have the Rpe65Met450 allele 148 and do not have the retinal degeneration 8 (rd8) allele (PCR genotype using Crb1 primers). Age of animals were listed in legends of each experiment. Mice used in the study were on a C57Bl/6 (Jackson) background with ages ranging from 1- 9 months.<br><br>Sex was not considered in the study design because this variable was not relevant to the study based on our studies showed that number of cSLO-detected AF foci is indistinguishable between females and males. Equal ratio of males and females mice were used for all the experiments.<br><br>All animals were housed in an animal facility at Weill Cornell Medicine at a relative constant temperature of 21.5±1°C and humidity of 30% to 70%, 12:12 hour light: dark photoperiod. Mice were housed in individually ventilated cages (Thoren Caging Systems, Hazelton, PA) on autoclaved aspen-chip bedding (PWI Industries Canada, Quebec, Canada); Cages are changed weekly in a HEPA-filtered vertical flow change station. γ-irradiated feed (LabDiet 5053, PMI, St Louis, MO, containing 24.495% protein,13.122% fat,62.382% Carbohydrates) and acidified reverse osmosis water (pH, 2.5 to 2.8) provided ad libitum.<br><br>All mice in these studies were euthanized by CO2 inhalation.
Wild animals	No wild animals were used in the study.
Reporting on sex	For the characterization and analysis of the new transgenic mice being reported in the manuscript, the study did not consider sex as a variable for present purposes. We use both genders randomly and roughtly equal ratio of males and females mice were used for all the experiments because our initial studies showed the number of AF foci detected by cSLO is indistinguishable between females and males. Data was not segregated based on sex for analysis.
Field-collected samples	No field-collected samples were used in the study.
Ethics oversight	All animal experiments were approved by Weill Cornell Medicine Institutional Animal Care and Use Committee (animal protocol 0605-490A).

Note that full information on the approval of the study protocol must also be provided in the manuscript.

Plants

Seed stocks	N/A
Novel plant genotypes	N/A
Authentication	N/A
